# A model for projecting individuals’ risk of esophageal squamous cell carcinoma in a high-risk Chinese population

**DOI:** 10.1093/gastro/goag049

**Published:** 2026-06-23

**Authors:** Liyan Zhao, Sihao Lin, Yi Shen, Xudong Liu, Yan Wang, Zhiqiang Liu, Fen Liu, Shao-Hua Xie

**Affiliations:** School of Public Health, Fujian Medical University, Fuzhou, Fujian, 350122, P. R. China; School of Management, Putian University, Putian, Fujian, 351100, P. R. China; Beijing Obstetrics and Gynecology Hospital, Capital Medical University, Beijing Maternal and Child Health Care Hospital, Beijing, 100006, P. R. China; School of Public Health, Guangdong Pharmaceutical University, Guangzhou, Guangdong, 510006, P. R. China; School of Public Health, Fujian Medical University, Fuzhou, Fujian, 350122, P. R. China; School of Public Health, Fujian Medical University, Fuzhou, Fujian, 350122, P. R. China; National Cancer Center/National Clinical Research Center for Cancer/Cancer Hospital, Chinese Academy of Medical Sciences and Peking Union Medical College, Beijing, 100021, P. R. China; School of Public Health, Capital Medical University, Beijing, 100069, P. R. China; School of Public Health, Fujian Medical University, Fuzhou, Fujian, 350122, P. R. China; Upper Gastrointestinal Surgery, Department of Molecular medicine and Surgery, Karolinska Institute, Stockholm, SE-17177, Sweden

**Keywords:** esophageal squamous cell carcinoma, risk prediction, prediction model, early detection, precision prevention

## Abstract

**Background:**

Early detection of esophageal squamous cell carcinoma (ESCC) may improve survival, but universal screening is not feasible. This study aimed to develop and validate a model for identifying individuals at high absolute risk of ESCC in a high-risk Chinese population.

**Methods:**

The model was developed by using data from a case–control study in Yanting County, Sichuan, China between 2011 and 2013, including 942 ESCC cases and 942 age- and sex-matched control participants. Conditional logistic regression and the Gail algorithm were used to construct the model. Model performance was assessed by using the area under the receiver-operating characteristic curve (AUC) with 10-fold cross-validation. External validation was performed in two independent populations, i.e. another Chinese case–control study and the UK Biobank cohort.

**Results:**

The model included six risk factors: education level, marital status, tobacco smoking, alcohol consumption, body mass index (overweight status), and family history of cancer. The model incorporated age- and sex-specific incidence rates in the population to estimate the 5-year absolute risk. The AUC was 0.72 (95% confidence interval [CI] 0.69–0.74) in the derivation dataset and 0.68 (95% CI, 0.67–0.69) after cross-validation. In external validation, the AUC was 0.65 (95% CI, 0.61–0.69) in the independent Chinese case–control study and 0.66 (95% CI, 0.56–0.75) in UK Biobank. The estimated 5-year absolute risk of ESCC in the population in Yanting ranged from 0.0005% to 18.2%. In the group at the highest predicted risk, six individuals would need to undergo screening to detect one ESCC case within 5 years.

**Conclusion:**

The developed model demonstrated acceptable performance and has the potential to identify high-risk individuals for targeted prevention and early detection in the studied high-risk population.

## Introduction

According to the Global Cancer Observatory, there were 511,000 new esophageal cancer cases and 445,000 related deaths worldwide in 2022, ranking esophageal cancer as the 11th-most common cancer type and the seventh leading cause of cancer-related mortality globally [1]. Esophageal squamous cell carcinoma (ESCC)—the predominant histological subtype of esophageal cancer—accounts for ∼90% of all esophageal cancer cases in low-income countries, particularly in Asia [2]. Despite the declining mortality rates in some regions over recent decades, China continues to bear more than half of the global burden of ESCC [3].

ESCC carries an extremely poor prognosis, with an estimated 5-year overall survival rate of only 10%–30% in Chinese populations [4]. Early detection may substantially reduce ESCC-related mortality. Its premalignant condition, esophageal squamous dysplasia, can be readily detected through endoscopy, offering an important opportunity for early intervention [5]. Previous studies in China have suggested that endoscopic screening in high-risk areas may reduce both the incidence and mortality of ESCC in the target population [6, 7]. However, the unselective endoscopic screening of the general population is not justified due to the low absolute risk of ESCC, as well as the high costs and procedure-related complications. Instead, identifying a limited group of individuals at high absolute risk for targeted endoscopic screening and surveillance represents a more feasible strategy.

Risk-prediction models based on readily identifiable risk factors offer a promising approach to identify individuals at high absolute cancer risk, enabling more practical and cost-effective prevention strategies [8–10]. Several risk-prediction models have been developed for ESCC, demonstrating fair to good discriminative accuracy, with reported area under the receiver-operating characteristic curve (AUC) values ranging from 0.71 to 0.88 [11]. However, few of these were developed in high-risk Chinese populations and have been externally validated.

By using data from a case–control study conducted in a high-risk area for ESCC in China, a risk-prediction model was developed to estimate individuals’ 5-year absolute risk of ESCC and validated the model in two independent populations.

## Methods

### Study design and participants

The model was developed in a case–control study conducted in Yanting County, Sichuan Province in Southwest China between 2011 and 2013. For external validation, data from another case–control study conducted in two high-risk areas in China and from a population-based cohort study in the UK (the UK Biobank cohort) were used. This study is reported in accordance with the Transparent Reporting of a multivariable prediction model for Individual Prognosis or Diagnosis (TRIPOD) guideline (Supplementary Table S1) [12].

The derivation dataset comprised 942 newly diagnosed ESCC cases recruited from the only local cancer hospital in Yanting County and 942 age- (±2 years) and sex-matched control participants randomly selected from the general population [13]. The hospital also houses the local cancer registry from which cancer incidence data are included in the “Cancer Incidence in Five Continents” series by the International Agency for Research on Cancer. According to the local cancer registry data, the included cases accounted for ∼70% of all incident ESCC cases in the county during the study period. There was no difference in age or gender distribution between the recruited and non-recruited cases in the county [13]. For each of the cases, one cancer-free control participant was selected by using the multistage sampling method from the general population, during which 6 townships were randomly selected first from 36 townships of Yanting County and control participants were randomly selected by using the resident identity card number for all eligible residents in the selected townships [13].

The external validation case–control study included 244 newly diagnosed ESCC cases and 1,220 control participants, recruited in Linzhou City, Henan Province (Central China) and Cixian County, Hebei Province (North China) between 2014 and 2016 [14]. The UK Biobank cohort is a prospective cohort that enrolled ∼500,000 participants aged 40–69 years from 22 recruitment centers across England, Scotland, and Wales during 2006–2010 [15, 16]. For the present analysis, 303,453 eligible participants who had no cancer diagnosis prior to cohort entry and no missing data on any of the selected predictors were included. These participants were followed until ESCC diagnosis, death, loss to follow-up, or the end of the study (31 July 2020), whichever occurred first. A total of 120 participants in UK Biobank developed ESCC during the follow-up, among whom 38 were diagnosed within 5 years. Additional details on these study populations are provided in Supplementary Method 1.

### Data collection

In the two Chinese case–control studies, participants were interviewed face to face in the local dialect by trained interviewers. A structured questionnaire was used to collect information on sociodemographic characteristics and potential ESCC risk factors. The specific questions and definitions for the key risk factors varied between studies and are described in detail in Supplementary Method 2. The case–control study for model derivation was approved by the Joint Chinese University of Hong Kong–New Territories East Cluster Clinical Research Ethics Committee. The case–control study for external validation was approved by the Ethics Committees of the Cancer Institute and Hospital, Chinese Academy of Medical Sciences, and Capital Medical University. Written consent form was obtained from all participants.

UK Biobank collected extensive phenotypic and genotypic data, including questionnaire-based information on risk factors, and objectively measured body weight and height. Participants are followed up for health conditions through linkage to national health datasets. All ESCC cases in UK Biobank were identified through linkage to the cancer registries by using histology codes according to the International Classification of Diseases for Oncology, 3rd edition (ICD-O-3), thereby excluding cases of esophageal adenocarcinoma. UK Biobank is regularly linked to UK cancer registry data from the Health and Social Care Information Centre (England and Wales) and the Scottish Cancer Registry (Scotland), and death records from the UK Office of National Statistics. UK Biobank was approved by the North West Multi-Centre Research Ethics Committee and all participants provided written informed consent.

### Predictors

The following six predictors were selected based on a literature review of risk factors for ESCC: education (<10 or ≥10 years), marital status (married/cohabiting or no), tobacco smoking (yes or no), daily alcohol drinking (yes or no), overweight status (yes or no), and family history of cancer in first-degree relatives (yes or no). These variables were prioritized because they have shown relatively consistent associations with ESCC risk in previous studies, were available in all the three datasets used in this study, and are easy to ascertain in clinical or public health settings. Although the questionnaires differed across the three studies, the data were harmonized by the application of consistent definitions for each risk predictor (Supplementary Method 2). Overweight was defined by using population-specific cutoffs of body mass index (Chinese criteria for the Chinese cohort and World Health Organization criteria for UK Biobank), reflecting differences in health risks related to body mass index across populations.

### Model development

Conditional logistic regression was used in the derivation dataset to assess the associations between the selected predictors and ESCC risk. For each predictor, both crude odds ratios (ORs) and ORs adjusted for all other selected predictors were estimated, together with 95% confidence intervals (CIs). Potential pairwise interactions between the predictor variables were examined by including cross-product terms into the logistic regression model. Because a statistically significant interaction was identified between daily alcohol drinking and overweight, these two factors were combined into a single predictor variable with four categories: (i) no daily alcohol drinking or overweight, (ii) no daily alcohol drinking and no overweight, (iii) daily alcohol drinking and overweight, and (iv) daily alcohol drinking and no overweight.

The Gail algorithm was used to estimate the absolute 5-year risk of ESCC for individuals with each possible risk-factor profile. This estimation was based on: (i) the estimated relative risk for the individual from the logistic model, (ii) the baseline age- and sex-specific incidence rate in the population, (iii) the estimated population-attributable risk derived from the logistic model, and (iv) the age- and sex-specific mortality rate in the population to correct for competing risk from death from causes other than esophageal cancer (Supplementary Table S1) [17, 18]. Details of the risk-calculation algorithm, as well as the specific incidence and competing mortality rates, are provided in Supplementary Methods 3.

### Assessment of model performance

The model performance was assessed by using the AUC and the Somers’ D statistics [19]. The AUC summarizes the discriminative ability of the model to distinguish ESCC cases from non-cases (control participants in case–control studies and individuals who did not develop ESCC in cohort studies). The Somers’ D statistic was used to assess the strength and direction of associations between the predicted probabilities and observed outcomes [19]. To account for possible over-fitting when the model performance was evaluated in the derivation dataset, 10-fold cross-validation was applied. In this procedure, the dataset was randomly partitioned into 10 subsets; in each iteration, 9 subsets were used to develop the model (training set) and the remaining subset was used for validation (test set). The final cross-validated performance estimates were obtained by averaging the results from the 10 iterations. The sensitivity, specificity, and predictive value of the full model were computed across the ESCC scores. The optimal score cutoff point for classifying individuals at high versus low risk of ESCC was selected based on three indexes: the Youden Index, defined as (sensitivity + specificity − 1); the distance to the ideal point (0, 1) in the AUC defined as the square root of ([1 − sensitivity]^2^ + [1 − specificity]^2^); and the sensitivity–specificity equality, defined as the absolute value of (sensitivity − specificity). A higher Youden index, a shorter distance to the ideal point, and lower sensitivity–specificity equality indicated better cutoff points [20].

The model performance was externally validated in two independent populations, i.e. the independent Chinese case–control study and the UK Biobank cohort. In UK Biobank, each participant’s 5-year absolute risk of ESCC was calculated by using the model, incorporating the age- and sex-specific incidence rates of ESCC and competing mortality rates obtained from the “Cancer Incidence in Five Continents” series and the United Kingdom Office for National Statistics (Supplementary Table S2) [17, 21]. Risk calibration in UK Biobank was assessed by plotting the observed cumulative proportions of ESCC cases against the predicted risks across deciles of predicted risks. A Hosmer–Lemeshow test was not performed because of the limited number (*n *= 38) of ESCC cases occurring within 5 years of follow-up.

All statistical analyses were performed by using the statistical software SAS version 9.4 (SAS Institute, Cary, NC) and R software (version 4.4.1). All *P* values were two-sided and a *P* value of <0.05 was considered statistically significant.

## Results

### Participants

The distributions of demographic characteristics and main risk factors in the derivation and validation populations are presented in Table 1. In the derivation case–control study, the mean age was 60 years in both the ESCC cases and control participants. In the Chinese validation case–control study, the mean ages were 61.7 (±7.8) years in ESCC cases and 59.1 (± 7.7) years in control participants. In UK Biobank, the mean age at cohort entry was 55.7 (± 8.1) years. A higher proportion of participants were males in the two case–control studies in China, with male-to-female ratios of 2.5:1 in the derivation study and 1.9:1 in the validation study. A smaller proportion of the ESCC cases (35.8%) were male in UK Biobank than in the entire cohort (46.4%).

**Table 1:** Distribution of characteristics of study participants in the derivation dataset and the validation datasets.

Variable	Yanting study		Linzhou and Cixian study		UK Biobank	
	Controls, *n* (%)	Cases, *n* (%)	Controls, *n* (%)	Cases, *n* (%)	Entire cohort, *n* (%)	Cases, *n* (%)
Total	942 (100)	942 (100)	1,220 (100)	244 (100)	299,708 (100)	38 (100)
Age, years						
40–49	103 (10.9)	104 (11.0)	175 (14.3)	20 (8.2)	79,271 (26.5)	4 (10.5)
50–59	311 (33.0)	319 (33.9)	367 (30.1)	66 (27.0)	104,784 (34.9)	8 (21.1)
≥60	528 (56.1)	519 (55.1)	678 (55.6)	158 (64.8)	115,653 (38.6)	84 (68.4)
Mean ± standard deviation	60.2 (6.8)	60.0 (6.8)	59.1 (7.7)	61.7 (7.8)	55.7 (8.1)	60.7 (6.8)
Sex						
Female	270 (28.7)	270 (28.7)	420 (34.4)	84 (34.4)	161,239 (53.8)	20 (52.6)
Male	672 (71.3)	672 (71.3)	800 (65.6)	160 (65.6)	138,469 (46.2)	18 (47.4)
Education, years						
≥10	17 (1.8)	15 (1.6)	25 (2.1)	3 (1.2)	257,397 (85.9)	31 (81.6)
<10	925 (98.2)	927 (98.4)	1,195 (97.9)	241 (98.8)	42,311 (14.1)	7 (18.4)
Marital status						
Married or cohabitating	847 (89.9)	884 (93.8)	1,136 (93.1)	212 (86.9)	269,556 (89.9)	32 (84.2)
Other	95 (10.1)	58 (6.2)	84 (6.9)	32 (13.1)	30,152 (10.1)	6 (15.8)
Tobacco smoking						
No	569 (60.4)	360 (38.2)	869 (71.2)	149 (61.1)	171,676 (57.3)	14 (36.8)
Yes	373 (39.6)	582 (61.8)	351 (28.8)	95 (38.9)	128,032 (42.7)	24 (63.2)
Daily alcohol drinking						
No	832 (88.3)	623 (66.2)	1,125 (92.2)	188 (77.0)	236,252 (78.8)	24 (63.2)
Yes	110 (11.7)	318 (33.8)	95 (7.8)	56 (23.0)	63,456 (21.2)	14 (36.8)
Overweight						
No	630 (66.9)	742 (78.7)	569 (46.6)	172 (70.5)	101,452 (33.8)	15 (39.5)
Yes	305 (32.4)	191 (20.3)	651 (53.4)	72 (29.5)	198,256 (66.2)	23 (60.5)
Missing	7 (0.7)	9 (1.0)	0	0	0	0
Family history of cancer						
No	749 (80.1)	692 (74.3)	874 (71.6)	150 (61.5)	188,882 (63.0)	23 (60.5)
Yes	186 (19.9)	240 (25.7)	346 (28.4)	94 (38.5)	110,826 (37.0)	15 (39.5)

### Conditional logistic regression

The distributions of the selected predictors and their associated ORs for ESCC in the derivation case–control study are shown in Table 2. Lower education level (<10 years), being married or cohabitating, tobacco smoking, daily alcohol drinking, and family history of cancer were associated with an increased risk of ESCC, while overweight was inversely associated with the risk of ESCC. Collectively, these six risk factors account for ∼80% of the ESCC cases in the derivation population (population-attributable fraction of 80%). The distributions of predictors and their association with ESCC risk in the two studies for external validation are shown in Supplementary Tables S3 and S4.

**Table 2:** ORs and 95% CIs for the associations between predictors and the risk of ESCC.

Variable	Controls, *n* (%)	Cases, *n* (%)	Crude OR (95% CI)	Adjusted OR (95% CI)
Education, years				
≥10	17 (1.82)	14 (1.50)	1.00 (reference)	1.00 (reference)
<10	918 (98.18)	917 (98.39)	1.21 (0.60–2.48)	1.52 (0.64–3.62)
Marital status				
Married or cohabitating	842 (90.05)	874 (93.78)	1.00 (reference)	1.00 (reference)
Other	93 (9.95)	58 (6.22)	0.60 (0.43–0.85)	0.52 (0.35–0.77)
Tobacco smoking				
No	564 (60.32)	356 (38.20)	1.00 (reference)	1.00 (reference)
Yes	371 (39.68)	576 (61.80)	2.46 (3.00–4.85)	3.26 (2.44–4.36)
Family history of cancer				
No	749 (80.11)	692 (74.25)	1.00 (reference)	1.00 (reference)
Yes	186 (19.89)	240 (25.75)	1.40 (1.12–1.74)	1.38 (1.07–1.77)
Alcohol drinking and overweight				
No daily alcohol drinking and overweight	263 (28.13)	137 (14.70)	1.00 (reference)	1.00 (reference)
No daily alcohol drinking and no overweight	562 (60.11)	481 (51.61)	1.64 (1.29–2.09)	1.85 (1.41–2.44)
Daily alcohol drinking and overweight	42 (4.49)	54 (5.79)	2.47 (1.57–3.88)	2.40 (1.44–3.98)
Daily alcohol drinking and no overweight	68 (7.27)	260 (27.90)	7.34 (5.24–10.29)	6.71 (4.48–10.05)

### Model performance and external validation

**Figure 1:**
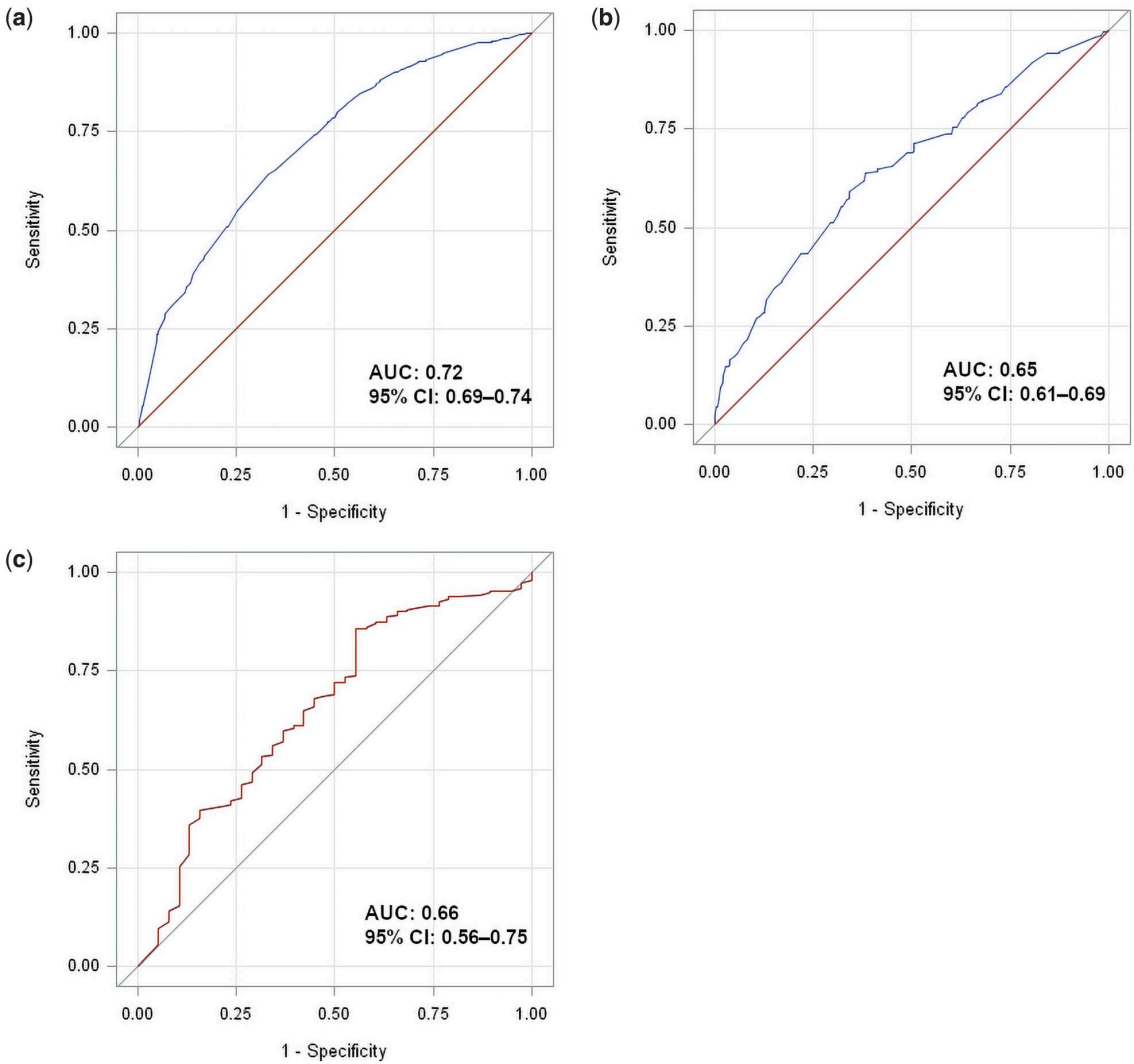
The receiver-operating characteristic curves for the risk-prediction model for ESCC (a) in the case–control study for model derivation, (b) in the case–control study for external validation, and (c) in UK Biobank for external validation.

**Figure 2:**
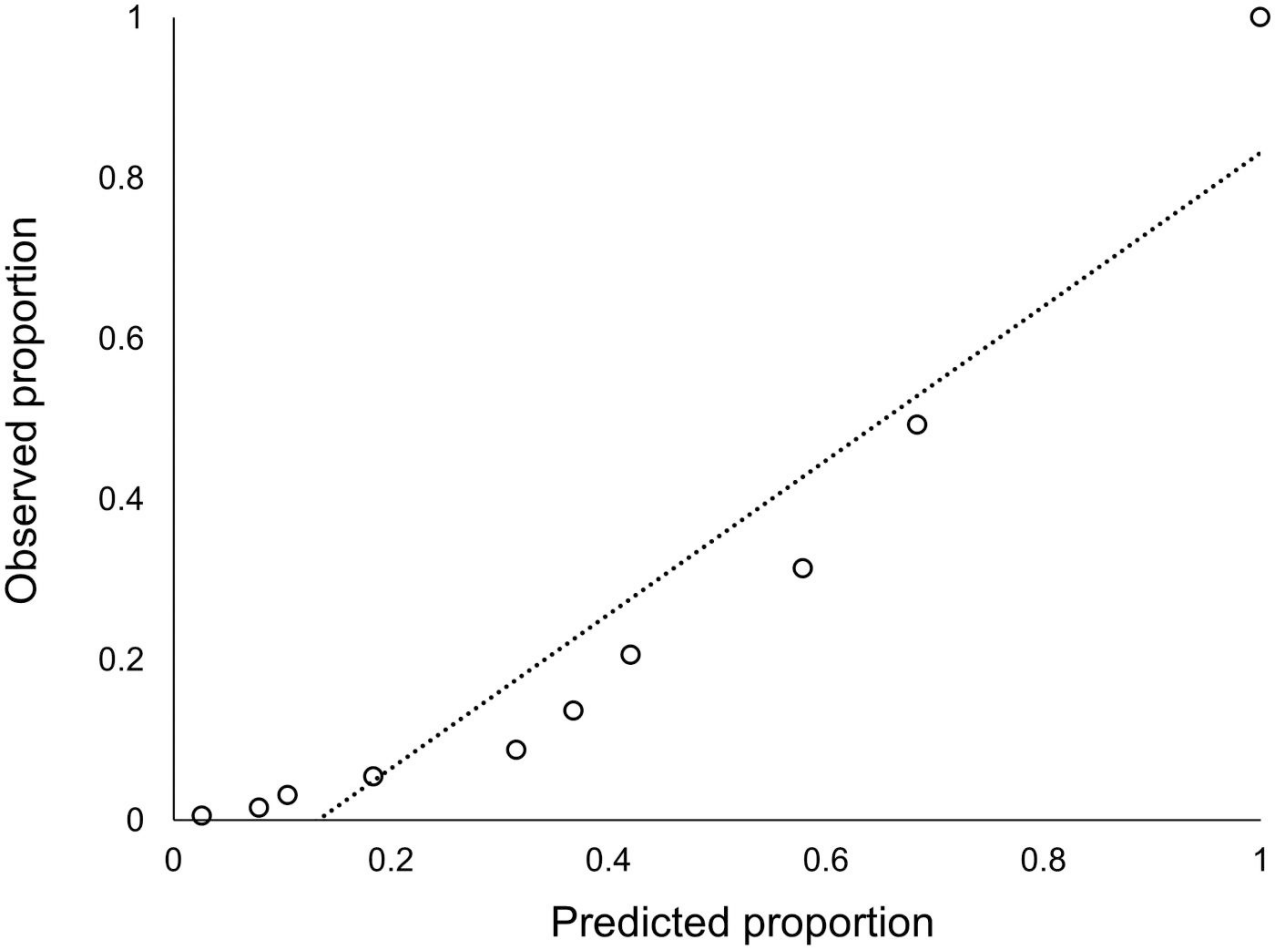
Calibration of the observed cumulative proportion of ESCC patients and predicted cumulative risks in the validation cohort (UK Biobank).

In the derivation data set, the AUC was 0.72 (95% CI, 0.69–0.74) and the Somers’ D statistic was 0.43. After 10-fold cross-valuation, the AUC decreased to 0.68 (95% CI, 0.67–0.69). In external validation, the AUC was 0.65 (95% CI, 0.61–0.69) in the independent Chinese case–control study and 0.66 (95% CI, 0.56–0.75) in UK Biobank (Table 3 and Fig. 1). The model also showed fair calibration in UK Biobank, with acceptable agreement between the predicted and observed risks (Fig. 2).

**Table 3:** Statistics for the discriminative accuracy of the risk-prediction model for ESCC in the derivation dataset and validation datasets.

Dataset	AUC (95% CI)	Somers’ D
Derivation dataset	0.72 (0.69–0.74)	0.43
Ten-fold cross-validation	0.68 (0.67–0.69)	0.36
External validation		
Linzhou and Cixian study	0.65 (0.61–0.69)	0.30
UK Biobank	0.66 (0.56–0.75)	0.31

To best discriminate high-risk persons from low-risk persons, the optimal cutoff analysis identified a maximum Youden index of 0.447 and a minimum distance to (0, 1) of 0.16 (Supplementary Table S5). At this cutoff, the model had a sensitivity of 76.82% and a specificity of 67.91%.

### Absolute 5-year risk of ESCC

The estimated 5-year absolute risks for ESCC for different combinations of risk factors ranged from as low as 0.0005% to as high as 18.2% in men and from 0.003% to 14.4% in women, as illustrated in a heat chart (Supplementary Fig. S1). Selected examples of the estimated 5-year absolute ESCC risks for specific risk-factor profiles are shown in Table 4. The highest 5-year absolute risk (18.2%) was predicted for a man aged 65–69 years with <10 years of education, who was married or cohabitating, a smoker and daily alcohol drinker, with a family history of cancer, and not overweight. In this group, approximately six individuals would need to undergo screening, for example through endoscopy, to detect one ESCC case within 5 years (Table 4).

**Table 4:** Estimated absolute 5-year risks for ESCC with selected profiles of risk factors.

Profile	Sex	Age, years	Education, years	Married/cohabitating	Tobacco smoking	Family cancer history	Daily alcohol drinking and overweight	Absolute 5-year risk, %	Number to survey to detect one case
1	Male	40–44	<10	No	No	No	No daily alcohol drinking and overweight	0.0005	200,000
2	Male	40–44	≥10	Yes	Yes	No	No daily alcohol drinking and overweight	0.1	1,000
3	Male	50–54	≥10	Yes	Yes	No	No daily alcohol drinking and overweight	0.7	143
4	Male	50–54	<10	Yes	Yes	No	No daily alcohol drinking and no overweight	1.4	72
5	Male	60–64	<10	No	Yes	Yes	Daily alcohol drinking and overweight	2.6	39
6	Male	60–64	<10	No	Yes	No	Daily alcohol drinking and no overweight	5.3	20
7	Male	65–69	<10	Yes	No	Yes	Daily alcohol drinking and no overweight	5.9	18
8	Male	65–69	<10	Yes	Yes	Yes	Daily alcohol drinking and no overweight	18.2	6
9	Female	40–44	≥10	No	No	No	No Daily alcohol drinking and overweight	0.003	33,334
10	Female	40–44	<10	Yes	No	Yes	No Daily alcohol drinking and overweight	0.1	1,000
11	Female	50–54	≥10	Yes	No	No	Daily alcohol drinking and overweight	0.4	250
12	Female	50–54	<10	Yes	No	Yes	Daily alcohol drinking and overweight	0.8	125
13	Female	60–64	≥10	No	Yes	Yes	Daily alcohol drinking and no overweight	6.7	13
14	Female	60–64	<10	Yes	Yes	Yes	Daily alcohol drinking and no overweight	14.4	7

## Discussion

A risk-prediction model was developed based on six readily identifiable risk factors to estimate individuals’ 5-year absolute risk of ESCC. The model showed fair to good discriminative accuracy in the derivation population, although its performance declined somewhat in external validation populations. Nevertheless, the model was able to identify subgroups of individuals at substantially elevated absolute risk of ESCC.

This study has several notable strengths. First, the model was developed in a study with broad population coverage and systematic recruitment procedures to minimize selection bias, capturing 70% of all incident ESCC cases in the study area during the study period, with randomly selected control participants from the general population and a high participation rate (96%) [13]. Second, the model performance was rigorously evaluated by using 10-fold cross-validation and external validation in two independent populations, thereby reduced the risk of over-fitting. Third, information on the predictors is readily available in similar studies or routine clinical settings, facilitating further external validation and potential implementation into practice in high-risk settings.

This study also has some limitations. First, as with any case–control study, misclassification of risk factors due to uncertainty in recalling past exposures was inevitable and might be differential between cases and control participants for certain exposures. However, the participants were unaware of the study hypotheses and trained non-medical interviewers used standardized data-collection protocols, which should have mitigated differential recall or reporting bias. Second, because cases and controls were matched on age and sex, these variables were not included in the logistic regression model, which might have led to underestimation of the discriminative performance metrics of the model, such as the AUC. Third, the inherent limitations of the case–control design precluded the assessment of risk calibration in the study for model derivation. Although the model was externally validated in UK Biobank as a prospective cohort, differences in the population risk profiles indicate that the ESCC risk-prediction models are likely population-specific and require local calibration before clinical use.

To our knowledge, only a limited number of prediction models have been developed for ESCC, most of which were developed in Chinese populations [14, 22–25]. These models have included varying sets of predictors. Four established risk factors, i.e. age, sex, tobacco smoking, and alcohol drinking, were included in all models, and family cancer history and body mass index were also frequently included. Some studies incorporated additional predictors, such as dietary factors; however, the evidence supporting these factors being causes of ESCC remains limited, and their prevalence and associated risk effects vary greatly across populations [26–28]. Notably, one previous model included alarm symptoms of ESCC as predictor, which may be problematic because the inclusion of alarm symptoms related to the predicted outcome may introduce over-adjustment or model over-fitting [25]. Another model, developed within an endoscopic screening program, included endoscopic findings (i.e. number of lesions, distinct lesions, and mild or moderate dysplasia) and reported high discriminative accuracy (AUC of 0.83 in the derivation dataset and 0.91 in the validation dataset) [24]. However, performing endoscopy solely to inform a risk-prediction model is not practical, even in that study, in which only 252 new cases of ESCC were identified during a follow-up of >400,000 person-years (incidence rate of 59.3 per 100,000 person-years). Only two studies in Western populations (a Swedish population-based case–control study [29] and a Norwegian population-based cohort study [20]) have developed risk-prediction models for ESCC, with the Norwegian model externally validated in UK Biobank [20, 29]. Although these models developed in Western populations showed lower AUCs than most of the available Chinese models, they were relatively simple and included only major established risk factors for ESCC. Overall, existing ESCC risk-prediction models differ greatly in study design and setting, population characteristics, included predictors, statistical analysis, and applicability, making direct comparisons challenging.

Importantly, only a small proportion of the previously published ESCC risk-prediction models have been externally validated. In our study, the AUC declined when applied to independent populations, despite the incorporation of age- and sex-specific incidence rates. The reduction in performance is likely attributable to differences in the definition and measurement of the predictors, as well as variations in the distribution and strength of association of risk factors across populations. For example, being married or cohabiting was associated with a higher ESCC risk in the derivation study, whereas the association was reversed in the other Chinese validation study and no clear association was observed in UK Biobank. Similarly, the association between daily alcohol drinking and the risk of ESCC was markedly stronger in the Chinese populations than in UK Biobank. Together, these discrepancies highlight the need for caution when applying a risk-prediction model to external populations with distinct risk-factor profiles.

The model developed in this study has valuable public health and clinical implications for high-risk Chinese populations. It relies on easily obtainable information on risk factors that can be collected through simple questionnaires or reviewing medical records. For individuals with the highest-risk profile identified by using our model (a 5-year risk of 18.2%), only six individuals would need to undergo endoscopic screening to detect one ESCC case. Conversely, the model may help to exclude individuals at low absolute risk of ESCC who are unlikely to benefit from screening, thereby avoiding unnecessary endoscopic examinations even in a high-risk region. Over the past two decades, several large-scale cancer screening programs have been launched in China, including endoscopic screening programs in selected regions with a high incidence of ESCC. Risk-prediction models, including the present one, could help to optimize these programs by enabling more targeted, effective, and sustainable strategies for ESCC prevention at a population level.

In summary, a model to predict individuals’ 5-year absolute risk of ESCC was developed based on readily identifiable risk factors and externally validated in two independent populations. The model showed acceptable performance and may facilitate the identification of high-risk individuals for targeted prevention and screening in the studied high-risk population.

## Supplementary Material

goag049_Supplementary_Data
