# Supplementary material for: A model for projecting individuals’ risk of esophageal squamous cell carcinoma in a high-risk Chinese population
Source: Gastroenterol Rep (Oxf). 2026 Jun 23;14:goag049. doi: 10.1093/gastro/goag049 (PMC13287996; doi:10.1093/gastro/goag049)
Supplement: goag049_Supplementary_Data [file goag049_supplementary_data.docx]

## Supplementary material

### Legends of supplementary materials

**Supplementary Methods 1.** Detailed description of data source.

**Supplementary Methods 2.** Definitions and assessment of the predictor variables.

**Supplementary Methods 3.** Estimation of absolute 5-year risks of esophageal squamous cell carcinoma.

**Supplementary Table S1.** TRIPOD checklist for prediction model development and validation

**Supplementary Table S2.** Age- and sex-specific incidence rates of esophageal squamous cell carcinoma and mortality rates excluding esophageal cancer in Yanting County, 2011-2013 (1/100 000)

**Supplementary Table S3.** Age- and sex-specific incidence rates of esophageal squamous cell carcinoma and mortality rates excluding esophageal cancer in the United Kingdom, 2006-2010 (1/100 000)

**Supplementary Table S4.** Odds ratios (OR) and 95% confidence intervals (CI) for the associations between predictors and the risk of esophageal squamous cell carcinoma in Linzhou and Cixian study

**Supplementary Table S5.** Odds ratios (OR) and 95% confidence intervals (CI) for the associations between predictors and the risk of esophageal squamous cell carcinoma in UK Biobank

**Supplementary Table S6.** Sensitivity and specificity of the risk prediction model for esophageal squamous cell carcinoma

**Supplementary Figure S1.** Heat chart for estimated absolute 5-year risk (%) of esophageal squamous cell carcinoma in (A) men and (B) women with various risk profiles.

**Supplementary Methods 1.** Detailed description of data source.

***Derivation dataset:*** A population-based case–control study was conducted in the Yanting County, China between June 2011 and May 2013. Newly diagnosed patients with esophageal squamous cell carcinoma (ESCC) were consecutively recruited from the local tumor hospital, which is the only specialty hospital for treating esophageal cancer in the county. Inclusion criteria for ESCC cases were individulas aged 40-70 years with newly diagnosed primary incident ESCC with a pathologic confirmation (ICD-10 code C15) and having lived in the area for >15 years. Among the 978 eligible cases, 942 (96%) cases participated in the study. According to the local cancer registry data, the included cases accounted for approximately 70% of all incident ESCC cases in the county during the study period. Of those nonrecruited ESCC cases, 98.5% were diagnosed by histopathology. There was no difference in age and gender distribution between the recruited and nonrecruited ESCC patients.

For each of the ESCC cases, one population-based control participant was selected with the multistage sampling method from the local residents who had lived in the county for at least 15 years. All control participants had no prior cancer diagnosis at any site. In selecting controls, six townships were randomly selected first from 36 townships of Yanting County. Sampling frame for all residents aged 40-70 years in the selected townships was available. Then, each control who matched with a case in terms of sex and age (within 2 years) was randomly selected, by using resident identity card number from the potentially eligible residents. If the first randomly selected resident did not agree to participate, the second randomly selected resident was approached. During the same period that cases were recruited, 886 (94%) people from the first round of selection and 56 (6%) from the second selection were recruited as individual matched controls. Finally, 942 pairs of cases and controls were included and analyzed in the study (1).

***Validation dataset 1:*** The case-control study for external validation study was based on a screening program for upper gastrointestinal cancer in Linzhou County of Henan Province and Cixian County of Hebei Province, China. A total of 20,000 participants were enrolled by a stratified cluster sampling procedure and participated in a baseline study on histopathology of the esophageal mucosa. All participants in the screening group were provided endoscopic examination by physicians who were trained by and under the supervision of experienced doctors from the National Cancer Center in China. The detailed study protocol was described in a previous study (2). The current study recruited 1,464 participants. They included 244 newly diagnosed ESCC cases: 42 cases from our early screening population, and 202 cases from the Endoscopy Center of Cancer Hospital, Linzhou County, Henan province, between 2014 and 2016. The participants were given careful endoscopic examinations of the entire esophagus and stomach. Then, suspicious biopsy specimens were obtained and read independently by two well-trained local pathologists. The histological diagnostic criteria were as previously described (3). The most severe diagnosis indicated by any of the biopsies was given as the global diagnosis for a participant. All ESCC cases in this study were diagnosed for the first time and pathologically confirmed. The stage and grade of ESCC were assessed according to the 7^th^ edition of the American Joint Committee on Cancer (AJCC) tumor-node-metastasis (TNM) staging system. A total of 1,220 eligible controls were randomly selected from our early 20,000 screening population. All the eligible controls are residents in the selected villages/communities, with no history of cancer or endoscopic examination in the latest 3 years, and are mentally and physically competent. All controls were frequency-matched by age (±5 years), sex, and residence area to the ESCC cases.

***Validation dataset 2:*** The UK Biobank includes participants recruited from 22 assessment centers in 2006-2010. Details of the cohort are presented in previous publications (4,5). In brief, 9.2 million British residents aged between 40 and 69 years who lived within 25 English miles of any of the 22 assessment centers and registered in the National Health Service were invited to participate. Among these, 502,628 (5.5%) participated and provided information regarding their lifestyle, medical history, and physical measures, and donated biological samples. The participants in the UK Biobank is representative of the general population in the United Kingdom regarding the distribution of age, sex and ethnicity, but not regarding lifestyle variables, physical measures, or health-related factors, possibly due to “healthy volunteer” selection bias (6). Information about cancer incidence and mortality is retrieved by linkages to the national cancer registries and national death registries in the United Kingdom.

**Supplementary Methods 2.** Definitions and assessment of the predictor variables.

**Derivation dataset:**

1. Tobacco smoking

Smoking was defined as lifetime consumption of ≥20 packs of cigarettes or daily smoking for ≥6 months, assessed via the question, “Did you smoke five years prior to diagnosis (cases) or interview (controls)?”

1. Daily alcohol drinking

Alcohol consumption was classified as monthly intake of ≥200 mL beer, ≥125 mL wine, or ≥50 mL spirits for ≥6 months, derived from responses to “Did you consume alcohol five years prior?” and “How frequently did you drink alcohol five years prior?”

1. Body mass index (BMI)

Subjects were asked to report their height and weight 5years before the interview. BMI was calculated as body weight in kilograms divided by the square of body height in meters (kg/m^2^), with overweight defined as BMI ≥24, defined by the Chinese industry standard Weight Determination for Adults [WS/T 428-2013].

1. Educational attainment

Educational attainment was categorized as ≥10 years (high school or above) or <10 years (junior high school, primary school, or less).

1. Marital Status

Participants were asked "What was your marital status 5 years ago?". Those who answered married were classified as married or cohabiting and those who answered unmarried or divorced or widowed were classified as other.

1. Family history of cancer

Participants were asked "Has anyone else in the family had cancer? " and "which relative? ". Having first-degree relatives with any cancer was classified as YES.

**Validation dataset 1:**

1. Tobacco smoking

Subjects were asked about their lifetime history of tobacco smoking. It included two options: non-smokers and smokers. Smokers referred to continuous smoking for more than six months with a self-estimated daily consumption of at least one cigarette. The non-smokers reported to have no current or previous history of tobacco use.

1. Daily alcohol drinking

Subjects were asked about their lifetime history of alcohol drinking. It included two options: non-drinkers and drinkers. Drinkers were defined as drinking at least one drink per day for more than 1 year. We categorized participants regarding whether they drink daily based on their answers to how much they drank every day.

1. BMI

Consistent with the derivation set.

1. Educational attainment

Consistent with the derivation set.

1. Marital Status

Consistent with the derivation set.

1. Family history of cancer

Subjects were asked about the history of cancers in the immediate family members and relatives within 3 generations. Individuals with at least one relative diagnosed with cancers were defined as having a family history of cancer. The participants were categorized into two groups: no and yes.

**Validation dataset 2:**

1. Tobacco smoking

Reconstructed from questionnaires of "Do you smoke tobacco now?" and "In the past, how often have you smoked tobacco?".

1. Daily alcohol drinking

Subjects were asked about "How often do you drink alcohol? ".

1. BMI

Calculated by the participant's weight in kg/(height in meter*height in meter) , with overweight defined as BMI ≥25.

1. Educational attainment

Subjects were asked about "Which of the following qualifications do you have? ". Participants who answered "none of the above" were categorized as <10 years.

1. Marital Status

Participants were asked "How are people in the household related to the participant?". Those who answered "Husband, wife or partner" were classified as married or cohabiting.

1. Family history of cancer

Participants were asked "illnesses of father, mother and siblings".

**Supplementary Methods 3.** Estimation of absolute 5-year risks of esophageal squamous cell carcinoma.

We calculated the absolute 5-year risks for all possible profiles of risk factors, based on the following information:

1. Relative risk for the individual

The relative risk associated with a specific profile of risk factors was calculated as the product of the odds ratios for individual risk factors.

2. Baseline age- and sex- specific incidence rates

We obtained the age- and sex-specific incidence rates of ESCC from the Cancer Incidence in Five Continents, which are presented in the supplementary table.

3. Population attributable risk of the model

The population attributable risk of the model was calculated by the following formula:

$$Population attributable risk=1-\frac{1}{x}\sum_{i=1}^{x} \left( \frac{1}{r_{i}} \right)$$

where $x$ was the number of ESCC cases, $r_{i}$ was the relative risk for the $i$th case estimated from the logistic regression model (*Bruzzi et al. Am J Epidemiol 1985;122:904-913*). (7)

4. Age- and sex-specific mortality rates excluding esophageal cancer

We calculated the age- and sex-specific mortality rates excluding esophageal cancer using the population mortality data from China Cause of Death Registration Data Set.

For an individual with the age of $t$ (in five-year groups), sex of $s$ (1=male, 2=female), and relative risk of $r$, we first calculated the baseline hazard as:

$$b_{1(t,s)}={IR}_{(t,s)}\times(1-population attributable risk)$$

where ${IR}_{(t,s)}$ was the age- and sex-specific incidence rate of ESCC in the population.

We estimated the absolute risk of ESCC over 5 years as:

$$P\left( t,s,r \right)=\frac{b_{1(t,s)}r}{b_{1(t,s)}r+b_{2(t,s)}}\times(1-e^{-5(b_{1(t,s)}r+b_{2(t,s)})})$$

where $b_{2(t,s)}$ was the age- and sex-specific mortality rate from competing causes.

**References**

1. Lin S, Wang X, Huang C, *et al.* Consumption of salted meat and its interactions with alcohol drinking and tobacco smoking on esophageal squamous-cell carcinoma. Int J Cancer 2015;137:582–589.

2. Chen W, Zeng H, Chen R, *et al.* Evaluating efficacy of screening for upper gastrointestinal cancer in China: a study protocol for a randomized controlled trial. Chin J Cancer Res 2017;29:294–302.

3. Wei W-Q, Hao C-Q, Guan C-T, *et al.* Esophageal Histological Precursor Lesions and Subsequent 8.5-Year Cancer Risk in a Population-Based Prospective Study in China. Am J Gastroenterol 2020;115:1036–1044.

4. Lyall DM, Celis-Morales C, Ward J, *et al.* Association of Body Mass Index With Cardiometabolic Disease in the UK Biobank: A Mendelian Randomization Study. JAMA Cardiol 2017;2:882–889.

5. Ganna A, Ingelsson E. 5 year mortality predictors in 498,103 UK Biobank participants: a prospective population-based study. Lancet Lond Engl 2015;386:533–540.

6. Fry A, Littlejohns TJ, Sudlow C, *et al.* Comparison of Sociodemographic and Health-Related Characteristics of UK Biobank Participants With Those of the General Population. Am J Epidemiol 2017;186:1026–1034.

7. Bruzzi P, Green SB, Byar DP, *et al.* Estimating the population attributable risk for multiple risk factors using case-control data. Am J Epidemiol 1985;122:904–914.

| **Supplementary Table S1.** TRIPOD checklist for prediction model development and validation | | | | |
| --- | --- | --- | --- | --- |
| **Section** | **Item** |  | **Checklist description** | **Page** |
| **Title and abstract** | | | | |
| Title | 1 | D;V | Identify the study as developing and/or validating a multivariable prediction model, the target population, and the outcome to be predicted. | 1 |
| Abstract | 2 | D;V | Provide a summary of objectives, study design, setting, participants, sample size, predictors, outcome, statistical analysis, results, and conclusions. | 1,2 |
| **Introduction** | | | | |
| Background and objectives | 3a | D;V | Explain the medical context (including whether diagnostic or prognostic) and rationale for developing or validating the multivariable prediction model, including references to existing models. | 3 |
|  | 3b | D;V | Specify the objectives, including whether the study describes the development or validation of the model or both. | 4 |
| **Methods** | | | | |
| Source of data | 4a | D;V | Describe the study design or source of data (e.g., randomized trial, cohort, or registry data), separately for the development and validation data sets, if applicable. | 5, Supplementary Method 1 |
|  | 4b | D;V | Specify the key study dates, including start of accrual; end of accrual; and, if applicable, end of follow-up. | 5, Supplementary Method 1 |
| Participants | 5a | D;V | Specify key elements of the study setting (e.g., primary care, secondary care, general population) including number and location of centres. | 5, 6, Supplementary Method 1 |
|  | 5b | D;V | Describe eligibility criteria for participants. | 5, Supplementary Method 1 |
|  | 5c | D;V | Give details of treatments received, if relevant. | Not applicable |
| Outcome | 6a | D;V | Clearly define the outcome that is predicted by the prediction model, including how and when assessed. | 5, 6, Supplementary Method 1 |
|  | 6b | D;V | Report any actions to blind assessment of the outcome to be predicted. | Not applicable |
| Predictors | 7a | D;V | Clearly define all predictors used in developing or validating the multivariable prediction model, including how and when they were measured. | 7, Supplementary Method 2 |
|  | 7b | D;V | Report any actions to blind assessment of predictors for the outcome and other predictors. | Not applicable |
| Sample size | 8 | D;V | Explain how the study size was arrived at. | 5 |
| Missing data | 9 | D;V | Describe how missing data were handled (e.g., complete-case analysis, single imputation, multiple imputation) with details of any imputation method. | 5, Supplementary Method 1 |
| Statistical analysis methods | 10a | D | Describe how predictors were handled in the analyses. | 7 |
|  | 10b | D | Specify type of model, all model-building procedures (including any predictor selection), and method for internal validation. | 7,8 |
|  | 10c | V | For validation, describe how the predictions were calculated. | 8,9 |
|  | 10d | D;V | Specify all measures used to assess model performance and, if relevant, to compare multiple models. | 8,9 |
|  | 10e | V | Describe any model updating (e.g., recalibration) arising from the validation, if done. | Not applicable |
| Risk groups | 11 | D;V | Provide details on how risk groups were created, if done. | Not applicable |
| Development vs. validation | 12 | V | For validation, identify any differences from the development data in setting, eligibility criteria, outcome, and predictors. | 7,8,9 |
| **Results** | | | | |
| Participants | 13a | D;V | Describe the flow of participants through the study, including the number of participants with and without the outcome and, if applicable, a summary of the follow-up time. A diagram may be helpful. | 10, Table 1 |
|  | 13b | D;V | Describe the characteristics of the participants (basic demographics, clinical features, available predictors), including the number of participants with missing data for predictors and outcome. | 10, Table 1 |
|  | 13c | V | For validation, show a comparison with the development data of the distribution of important variables (demographics, predictors and outcome). | 10, Table 1 |
| Model development | 14a | D | Specify the number of participants and outcome events in each analysis. | 10, Table 2 |
|  | 14b | D | If done, report the unadjusted association between each candidate predictor and outcome. | Table 2 |
| Model specification | 15a | D | Present the full prediction model to allow predictions for individuals (i.e., all regression coefficients, and model intercept or baseline survival at a given time point). | 11, Table 4 |
|  | 15b | D | Explain how to the use the prediction model. | 11 Table 4 |
| Model performance | 16 | D;V | Report performance measures (with CIs) for the prediction model. | 10,11, Table 3 and Figure 1 |
| Model-updating | 17 | V | If done, report the results from any model updating (i.e., model specification, model performance). | Not applicable |
| **Discussion** | | | | |
| Limitations | 18 | D;V | Discuss any limitations of the study (such as nonrepresentative sample, few events per predictor, missing data). | 12,13 |
| Interpretation | 19a | V | For validation, discuss the results with reference to performance in the development data, and any other validation data. | 13,14 |
|  | 19b | D;V | Give an overall interpretation of the results, considering objectives, limitations, and results from similar studies, and other relevant evidence. | 13,14,15 |
| Implications | 20 | D;V | Discuss the potential clinical use of the model and implications for future research. | 15 |
| **Other information** | | | | |
| Supplementary information | 21 | D;V | Provide information about the availability of supplementary resources, such as study protocol, Web calculator, and data sets. | Supplementary |
| Funding | 22 | D;V | Give the source of funding and the role of the funders for the present study. | 16 |

* Items relevant only to the development of a prediction model are denoted by D, items relating solely to a validation of a prediction model are denoted by V, and items relating to both are denoted D;V. We recommend using the TRIPOD Checklist in conjunction with the TRIPOD Explanation and Elaboration document.

| **Supplementary Table S2.** Age- and sex-specific incidence rates of esophageal squamous cell carcinoma and population mortality rates excluding esophageal cancer in Yanting County, 2011–2013 (1/100,000) | | | | | | |
| --- | --- | --- | --- | --- | --- | --- |
| **Age, years** | **Male** | | |  | **Female** | |
|  | **Incidence** | **Mortality** | |  | **Incidence** | **Mortality** |
| 30–34 | 6.7 | | 190.3 |  | 3.5 | 71.6 |
| 35–39 | 25.4 | | 223.3 |  | 4.0 | 84.1 |
| 40–44 | 34.6 | | 337.8 |  | 39.7 | 141.6 |
| 45–49 | 88.3 | | 488.3 |  | 87.2 | 217.9 |
| 50–54 | 165.0 | | 661.0 |  | 126.3 | 323.2 |
| 55–59 | 226.9 | | 949.4 |  | 134.0 | 478.9 |
| 60–64 | 297.7 | | 1575.0 |  | 317.1 | 879.0 |
| 65–69 | 424.0 | | 2205.3 |  | 289.3 | 1340.5 |
| 70–74 | 719.6 | | 3767.6 |  | 334.3 | 2400.6 |
| 75–79 | 577.3 | | 6172.0 |  | 230.4 | 4057.2 |
| 80–84 | 1322.2 | | 10989.2 |  | 371.7 | 7532.3 |
| 85+ | 815.0 | | 24819.1 |  | 421.9 | 18277.7 |

| **Supplementary Table 3.** Age- and sex-specific incidence rates of esophageal squamous cell carcinoma and mortality rates excluding esophageal cancer in the United Kingdom, 2006-2010 (1/100 000) | | | | | | |
| --- | --- | --- | --- | --- | --- | --- |
| **Age, years** | **Male** | |  | **Female** | | |
|  | **Incidence** | **Mortality** |  | **Incidence** | | **Mortality** |
| 30–34 | 0 | 100.1 |  | 0 | 47.1 | |
| 35–39 | 0.4 | 130.0 |  | 0.1 | 70.5 | |
| 40–44 | 0.5 | 177.5 |  | 0.5 | 112.4 | |
| 45–49 | 1.1 | 266.7 |  | 1.1 | 176.6 | |
| 50–54 | 2.0 | 251.0 |  | 1.8 | 288.5 | |
| 55–59 | 4.2 | 395.0 |  | 2.3 | 439.9 | |
| 60–64 | 6.1 | 605.6 |  | 2.7 | 692.3 | |
| 65–69 | 9.5 | 996.1 |  | 5.9 | 1,132.6 | |
| 70–74 | 13.8 | 1,574.9 |  | 9.8 | 1,970.0 | |
| 75–79 | 15.3 | 2,588.0 |  | 10.6 | 3,783.7 | |
| 80–84 | 15.6 | 4,116.6 |  | 9.6 | 7,668.7 | |
| 85+ | 33.5 | 5,661.5 |  | 16.3 | 15,300.7 | |

| **Supplementary Table 4. Odds ratios (OR) and 95% confidence intervals (CI) for the associations between predictors and the risk of esophageal squamous cell carcinoma in Linzhou and Cixian study** | | | | |
| --- | --- | --- | --- | --- |
| **Variable** | **Controls, n (%)** | **Cases, n (%)** | **Crude OR (95%CI)** | **Adjusted OR (95%CI)** |
| Education |  |  |  |  |
| ≥10 years | 25 (2.1) | 3 (1.2) | 1.00 (reference) | 1.00 (reference) |
| <10 years | 1,195 (97.9) | 241 (98.8) | 1.68 (0.50–5.61) | 1.47 (0.42–5.18) |
| Marital Status |  |  |  |  |
| Married or cohabitating | 1,136 (93.1) | 212 (86.9) | 1.00 (reference) | 1.00 (reference) |
| Other | 84 (6.9) | 32 (13.1) | 2.04 (1.32–3.15) | 2.38 (1.50–3.77) |
| Tobacco smoking |  |  |  |  |
| No | 869 (71.2) | 149 (61.1) | 1.00 (reference) | 1.00 (reference) |
| Yes | 351 (28.8) | 95 (38.9) | 1.58 (1.19–2.10) | 1.20 (0.87–1.65) |
| Family history of cancer |  |  |  |  |
| No | 874 (71.6) | 150 (61.5) | 1.00 (reference) | 1.00 (reference) |
| Yes | 346 (28.4) | 94 (38.5) | 1.58 (1.19–2.11) | 1.48 (1.10–2.00) |
| Alcohol drinking and overweight |  |  |  |  |
| No daily alcohol drinking and overweight | 592 (48.5) | 57 (23.3) | 1.00 (reference) | 1.00 (reference) |
| No daily alcohol drinking and no overweight | 533 (43.7) | 131 (53.7) | 2.55 (1.83–3.56) | 2.62 (1.87–3.66) |
| Daily alcohol drinking and overweight | 59 (4.8) | 15 (6.2) | 2.64 (1.41–4.95) | 2.65 (1.38–5.10) |
| Daily alcohol drinking and no overweight | 36 (3.0) | 41 (16.8) | 11.83 (7.00–19.97) | 11.55 (6.66–20.02) |

| **Supplementary Table 5. Odds ratios (OR) and 95% confidence intervals (CI) for the associations between predictors and the risk of esophageal squamous cell carcinoma in UK Biobank** | | | | |
| --- | --- | --- | --- | --- |
| **Variable** | **Entire cohort, n (%)** | **Cases, n (%)** | **Crude OR (95%CI)** | **Adjusted OR (95%CI)** |
| Education |  |  |  |  |
| ≥10 years | 257,397 (85.9) | 31 (81.6) | 1.00 (reference) | 1.00 (reference) |
| <10 years | 42,311 (14.1) | 7 (18.4) | 1.37 (0.61–3.12) | 1.39 (0.60–3.18) |
| Marital Status |  |  |  |  |
| Married or cohabitating | 269,556 (89.9) | 32 (84.2) | 1.00 (reference) | 1.00 (reference) |
| Other | 30,152 (10.1) | 6 (15.8) | 1.68 (0.70–4.01) | 1.75 (0.73–4.20) |
| Tobacco smoking |  |  |  |  |
| No | 171,676 (57.3) | 14 (36.8) | 1.00 (reference) | 1.00 (reference) |
| Yes | 128,032 (42.7) | 24 (63.2) | 2.30 (1.19–4.44) | 2.07 (1.06–4.06) |
| Family history of cancer |  |  |  |  |
| No | 188,882 (63.0) | 23 (60.5) | 1.00 (reference) | 1.00 (reference) |
| Yes | 110,826 (37.0) | 15 (39.5) | 1.11 (0.58–2.13) | 1.08 (0.56–2.07) |
| Alcohol drinking and overweight |  |  |  |  |
| No daily alcohol drinking and overweight | 158,109 (52.7) | 15 (39.5) | 1.00 (reference) | 1.00 (reference) |
| No daily alcohol drinking and no overweight | 78,143 (26.1) | 9 (23.7) | 1.21 (0.53–2.77) | 1.31 (0.57–3.01) |
| Daily alcohol drinking and overweight | 40,147 (13.4) | 8 (21.0) | 2.10 (0.89–4.96) | 1.95 (0.82–4.65) |
| Daily alcohol drinking and no overweight | 223,309 (7.8) | 6 (15.8) | 2.71 (1.05–6.99) | 2.65 (1.02–6.90) |

| **Supplementary Table 6.** Sensitivity and specificity of the risk prediction model for esophageal squamous cell carcinoma | | | | | | | | | | | |
| --- | --- | --- | --- | --- | --- | --- | --- | --- | --- | --- | --- |
| Risk threshold | High risk count | High risk pct (%) | True positive (count) | False positive (count) | True negative (count) | False negative (count) | Sensitivity (%) | Specificity (%) | Youden index | Distance to (0,1) | Sensitivity -specificity equality |
| 0.01 | 1,850 | 99.09 | 925 | 925 | 10 | 7 | 99.25 | 1.07 | 0.003 | 0.98 | 0.98 |
| 0.05 | 1,822 | 97.59 | 922 | 900 | 35 | 10 | 98.93 | 3.74 | 0.027 | 0.93 | 0.95 |
| 0.1 | 1,735 | 92.93 | 916 | 819 | 116 | 16 | 98.28 | 12.41 | 0.107 | 0.77 | 0.86 |
| 0.15 | 1,653 | 88.54 | 908 | 745 | 190 | 24 | 97.42 | 20.32 | 0.177 | 0.64 | 0.77 |
| 0.2 | 1,592 | 85.27 | 901 | 691 | 244 | 31 | 96.67 | 26.10 | 0.228 | 0.55 | 0.71 |
| 0.25 | 1,459 | 78.15 | 866 | 593 | 342 | 66 | 92.92 | 36.58 | 0.295 | 0.41 | 0.56 |
| 0.3 | 1,371 | 73.43 | 843 | 528 | 407 | 89 | 90.45 | 43.53 | 0.34 | 0.33 | 0.47 |
| 0.35 | 1,330 | 71.24 | 824 | 506 | 429 | 108 | 88.41 | 45.88 | 0.343 | 0.31 | 0.43 |
| 0.4 | 1,212 | 64.92 | 785 | 427 | 508 | 147 | 84.23 | 54.33 | 0.386 | 0.23 | 0.30 |
| 0.45 | 1,085 | 58.11 | 731 | 354 | 581 | 201 | 78.43 | 62.14 | 0.406 | 0.19 | 0.16 |
| **0.5** | **1,016** | **54.42** | **716** | **300** | **635** | **216** | **76.82** | **67.91** | **0.447** | **0.16** | **0.09** |
| 0.55 | 765 | 40.97 | 571 | 194 | 741 | 361 | 61.27 | 79.25 | 0.405 | 0.19 | 0.18 |
| 0.6 | 638 | 34.17 | 498 | 140 | 795 | 434 | 53.43 | 85.03 | 0.385 | 0.24 | 0.32 |
| 0.65 | 520 | 27.85 | 419 | 101 | 834 | 513 | 44.96 | 89.20 | 0.342 | 0.31 | 0.44 |
| 0.7 | 479 | 25.66 | 397 | 82 | 853 | 535 | 42.60 | 91.23 | 0.338 | 0.34 | 0.49 |
| 0.75 | 391 | 20.94 | 332 | 59 | 876 | 600 | 35.62 | 93.69 | 0.293 | 0.42 | 0.58 |
| 0.8 | 258 | 13.82 | 234 | 24 | 911 | 698 | 25.11 | 97.43 | 0.225 | 0.56 | 0.72 |
| 0.85 | 197 | 10.55 | 180 | 17 | 918 | 752 | 19.31 | 98.18 | 0.175 | 0.65 | 0.79 |
| 0.9 | 115 | 6.16 | 106 | 9 | 926 | 826 | 11.37 | 99.04 | 0.104 | 0.79 | 0.88 |
| 0.95 | 28 | 1.50 | 25 | 3 | 932 | 907 | 2.68 | 99.68 | 0.024 | 0.95 | 0.97 |


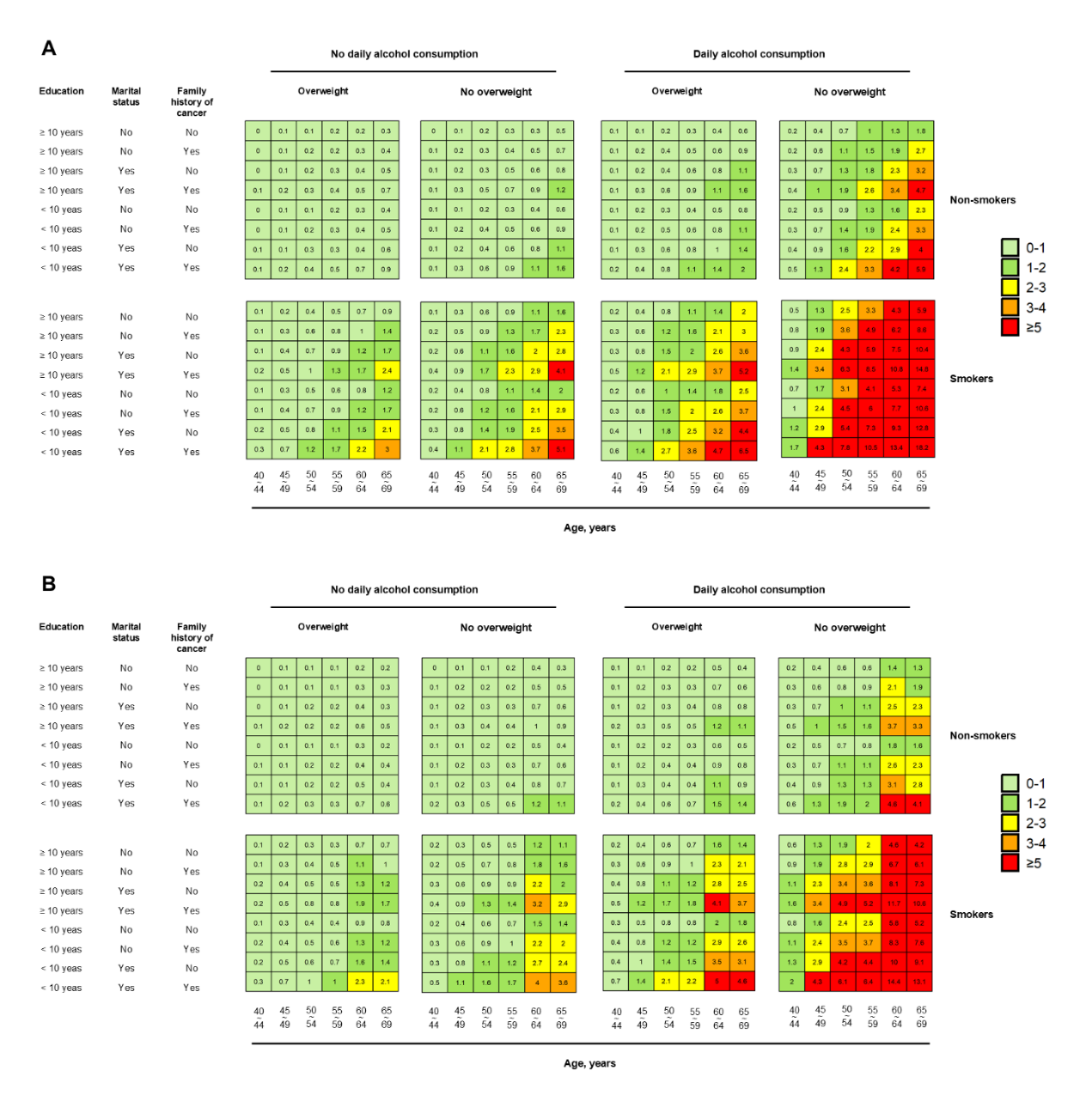


**Supplementary Figure S1.** Heat chart for estimated absolute 5-year risk (%) of esophageal squamous cell carcinoma in (A) men and (B) women with various risk profiles
